# Detection of antibodies against influenza D virus in swine veterinarians in Italy in 2004

**DOI:** 10.1002/jmv.27466

**Published:** 2021-11-27

**Authors:** Claudia Maria Trombetta, Emanuele Montomoli, Ilaria Di Bartolo, Fabio Ostanello, Chiara Chiapponi, Serena Marchi

**Affiliations:** ^1^ Department of Molecular and Developmental Medicine University of Siena Siena Italy; ^2^ VisMederi srl Siena Italy; ^3^ VisMederi Research srl Siena Italy; ^4^ Department of Food Safety, Nutrition and Veterinary Public Health Istituto Superiore di Sanità Rome Italy; ^5^ Department of Veterinary Medical Sciences University of Bologna Bologna Italy; ^6^ Sede territoriale di Parma, OIE Reference Laboratory for Swine Influenza Istituto Zooprofilattico Sperimentale della Lombardia ed Emilia Romagna Brescia Italy

**Keywords:** influenza D viruses, Italy, veterinarians

## Abstract

Influenza D virus (IDV) was first isolated from a swine with respiratory disease symptoms in 2011 in the United States. Epidemiological and serological studies support the hypothesis that cattle represent the natural reservoir of IDV with periodical spillover events to other animal hosts. Little is known about the seroprevalence in humans and in specific target groups such as veterinarians in Italy. This study was designed to assess the prevalence of antibodies against two influenza D lineages (D/660 and D/OK) in Italy in archived serum samples from veterinarians working with swine collected in 2004. Serum samples were tested by haemagglutination inhibition (HI) and virus neutralization (VN) assays. Results showed that 4.88% (4/82) of tested samples were positive for D/660 and 2.44% (2/82) for D/OK by HI assay. Three out of four samples showed positivity when tested by VN assay. Our data suggest undetected IDVs might have circulated and/or been introduced in Italy as early as 2004 at least in some animal species such as swine. In addition, it seems that the virus was circulating among veterinarians before the first isolation in 2011. This finding highlights the importance to continue monitoring the IDV spread in animals and humans for more detailed surveillance.

## 1 INTRODUCTION

Influenza D virus (IDV) is a novel influenza virus isolated from a swine with respiratory disease symptoms in 2011 in the United States.^1^ IDV, as influenza C virus (ICV), has seven RNA segments and only one major surface glycoprotein, the hemagglutinin‐esterase‐fusion, which is responsible for binding, receptor destroying, and fusion. The homology of amino acid sequences between IDV and ICV is roughly 50%, however, the distance between the two influenza viruses is similar to the one found between influenza A and B viruses (IAVs, IBVs).^2^, ^3^ No cross‐reactivity has been detected between IDV and ICV.^4^

Although the virus has been first isolated in swine, several epidemiological and serological studies support the hypothesis that cattle represent the natural reservoir of IDV with periodical spillover events to other animal hosts (i.e., camel, sheep, swine, horse, goat).^4^, ^5^, ^6^ The viral genome has been detected in some animal species while only specific antibodies have been detected in horses without evidence of viral genome or virus isolation.^7^ So far, there is no evidence of IDV infection in chickens and turkeys.^8^ The virus has been identified in different countries across the world (i.e., France, Italy, Luxemburg, Canada, Mexico, China, Mississippi, Japan, Nebraska).^5^, ^6^, ^9^, ^10^, ^11^, ^12^

To date, different lineages have been identified, D/OK‐, D/660‐, D/Japan‐lineages (D/Yama2016 and D/Yama2019) and the recently identified D/CA2019.^13^ D/OK and D/660 are the two major circulating lineages in North America and Europe. Until 2017, the only circulating lineage in Italy was D/OK, after phylogenetic analysis, the D/660 has been detected showing co‐circulation of both lineages in the Italian cattle population.^14^

IDV seroprevalence in different animal species has been assessed in Italy. Cattle show to have a high prevalence ranging from 92.4% to 74% (active and active/passive surveillance, respectively). Regarding swine from Northern Italy, the seroprevalence value was from 0.6% to 11.7% depending on the year of sampling (2009–2018). Low prevalence has been detected in wild boars from the Alpine and Northern Apennine areas (1.92%, 2018–2019) and in wild ungulates (0.98%). Sheep and goats show a prevalence of 6.3% and 3.1%, respectively, in 2016–2017.^15^, ^16^

The live trade seems to play a key role in viral spread considering that Italy, together with Spain, is one of the most important cattle importers in Europe from France. Data report higher IDV seroprevalence in importing countries (i.e. Italy) than in exporting countries suggesting that cattle may come in contact with the virus during transportation or just after.^15^

IDV seroprevalence in Italy has been studied on general population only, ranging from 5.1% in 2005 to 46.0% in 2014.^17^ International studies on cattle and farming workers performed in Florida and Malaysia showed a seroprevalence of 94% and 1.3%, respectively, suggesting that cattle‐exposed people could be infected with IDV through occupational zoonotic transmission.^18^, ^19^

The aim of this study was to assess the prevalence of antibodies against two IDV lineages (D/660 and D/OK) in Italy in archived serum samples from veterinarians working with swine collected in 2004.

## 2 MATERIALS AND METHODS

### 2.1 Influenza viruses

Influenza D/bovine/Oklahoma/660/2013 (D/660) and influenza D/OK ‐D/swine/Italy/199724/2015 (D/OK) viruses were propagated in Madin‐Darby Canine Kidney (MDCK) cells as previously described.^17^

### 2.2 Serum samples

A total of 82 serum samples were collected from a group of Italian veterinarians working with swine and attending the 30^th^ meeting of the Italian Society of Pathology and Breeding of Pigs (SIPAS) in 2004. The enrolled veterinarians worked in Northern and Central Italy, an area with the highest density of pigs and swine farms.

The median age of the study population was 41 years, with a range of 24–76 years; 76.8% of samples were from male subjects.

Influenza D (swine) hyperimmune serum against D/swine/Italy/199724/2015 was used as the positive control.

Influenza C, influenza A (H1N1 and H3N2), influenza B (Victoria and Yamagata lineages) hyperimmune serum samples were included as controls in the assay.

Human serum without immunoglobulin A, immunoglobulin M, and immunoglobulin G was used as a negative control (Sigma‐Aldrich).

All serum samples were tested by the haemagglutination inhibition (HI) assay.

Positive samples with a sub‐set of negative samples were tested by the virus neutralization (VN) assay.

### 2.3 HI assay

The HI assay was performed as previously described.^17^ All serum samples, including positive and negative controls, were pretreated with receptor‐destroying enzyme (ratio 1:5) from Vibrio cholerae (Sigma‐Aldrich) for 18 h at 37°C in a water bath followed by heat inactivation for 1 h at 56°C in a water bath with 8% sodium citrate (ratio 1:4). All serum samples were tested in duplicate using turkey red blood cells (0.35%). The antibody titer was expressed as the reciprocal of the highest serum dilution showing complete inhibition of agglutination. Since the starting dilution was 1:10, a titer below the detectable threshold was conventionally expressed as 5 for calculation purposes.

### 2.4 Virus neutralization

The MDCK cell cultures were grown at 37°C in 5% CO_2_ and pre‐incubated for 4 h.

Serum samples, including positive and negative controls, were previously heat‐inactivated at 56°C for 30 min. Samples twofold diluted with EMEM culture medium supplemented with 0.5% fetal bovine serum were mixed with an equal volume of virus (100 TCID50/well). After 1 h of incubation at 37°C in 5% CO_2_, 100 µl of the mixture was transferred to a plate containing 1.5 × 10^4^ MDCK cells/well. Plates were read for haemagglutination activity in the supernatant after 5 days of incubation at 37°C in 5% CO_2_. The VN titer was expressed as the reciprocal of the highest serum dilution showing the absence of haemagglutination.

### 2.5 Statistical analysis

Seroprevalence rates were calculated along with the corresponding 95% confidence interval (CI) using the adjusted Wald method (GraphPad QuickCalc, https://www.graphpad.com/quickcalcs/).

## 3 RESULTS

Out of 82 samples tested, 4 samples (4.88%, 95% CI: 1.54–12.26) showed HI positivity for D/bovine/Oklahoma/660/2013 strain (D/660‐lineage), two of them (2.44%, 94% CI: 0.15–8.98) were positive for D/swine/Italy/199724/2015 strain (D/OK‐lineage) as well. The HI levels of positivity range from 10 to 80 for D/660 and from 20 to 80 for D/OK (Table 1).

**Table 1:** HI and VN titers of veterinarians samples collected in 2004 by tested lineage

		HI assay		VN assay	
Sample	Age (years)	D/660	D/OK	D/660	D/OK
1	42	10–10	5	40	20
2	28	80–40	5	40	10
3	39	80–80	80–80	ND	ND
4	50	40–80	20–20	40	20

Abbreviations: HI, haemagglutination inhibition; ND, not determined, VN, virus neutralization.

Three out of 4 samples were further tested by the VN assay and showed positivity for both lineages with titers ranging from 10 to 40 (Table 1). Unfortunately, we did not have enough serum for one sample to be tested by the VN assay (Table 1).

Table 2 shows the HI titers of all controls against D/bovine/Oklahoma/660/2013 strain (D/660‐lineage) and D/swine/Italy/199724/2015 strain (D/OK‐lineage).

**Table 2:** HI titers of hyperimmune serum samples included as controls in the assay

Assay control	IDV lineages	
	D/660	D/OK
IDV (D/OK)	1280	10240
ICV	5	5
H1N1	5	5
H3N2	5	5
B Victoria	5	5
B Yamagata	5	5

*Note*: IDV: hyperimmune serum against D/swine/Italy/199724/2015 virus; ICV: hyperimmune serum against C/Victoria/2/2012 virus; H1N1: hyperimmune serum against A/Michigan/45/2015 (H1N1) virus; H3N2: hyperimmune serum against A/Hong Kong/45/2015 (H3N2) virus; B Victoria: hyperimmune serum against B/Brisbane/60/2008 virus; B Yamagata: hyperimmune serum against B/Phuket/3073/2013 virus.

## 4 DISCUSSION

In this study, we tested 82 archived serum samples from swine veterinarians, working in Northern and Central Italy, collected in 2004 and 4.8% had HI titers ≥10 for D/660. This positive result suggested that there might have been undetected IDV circulation or introduction in Italy as early as 2004. Our previous study conducted in Italy from 2005 to 2017 has shown that 5.1% of the general population had antibodies against D/660 in 2005. International studies have detected IDV or antibodies against IDV in cattle workers in Florida in 2011–2012,^18^ in humans recruited in Canada and Connecticut during 2007–2008 and 2008–2009 influenza seasons,^1^ in animal workers in Malaysia in 2017,^19^ in bioaerosol sampling in North Carolina in 2017–2018 and in 2018.^20^, ^21^ Notably, two studies performed in animals revealed antibodies specific for IDV in one goat sample collected in April 2002 in Massachusetts and in the Mississippi cattle population since at least 2004,^8^, ^9^ highlighting the possibility that the virus was circulating among some animal species before the first isolation in 2011.

The HI‐positive samples were further tested by the VN assay. All samples positive for D/660 were confirmed by the VN assay. Surprisingly, two samples tested negative by HI for D/OK, showed measurable antibody titers, though low, by the VN assay. The same inconsistency has been detected in another study^8^ conducted in animal serum samples providing two possible explanations. The first one might be the more sensitive nature of the VN assay for detecting antibodies. The other one could be the ability of the VN assay to detect functional antibodies different from the ones detected by the HI assay. Overall, based on the VN results, the seroprevalence provided by the HI assay may be slightly underestimated.

Based on the HI and VN data, we found human samples positive for both lineages, D/660 and D/OK. Serological data on animal samples found low positivity for D/OK in 2009 and a steady increase from roughly 2015 in Italy.^22^ In addition, it seems that up to 2017, all the Italian IDVs isolated belonged to the D/OK genetic cluster and the earliest D/660 strains were detected in 2018 from cattle imported from France.^14^ These findings might appear to be in contrast with our results. However, it should be pointed out that further investigations on animal samples, sera, and swabs, coming from different animal species and geographic areas, are needed to better understand and explore IDVs circulation and/or introduction in Italy. We can hypothesize undetected introduction of D/660 in animals, particularly in swine based on our data, with an undetected animal outbreak and that maybe the virus has started to steadily circulate in recent years only. On the other hand, as the VN titers for D/OK are slightly lower than those for D/660, it is possible that the exposition to one IDV can induce cross‐reactive antibodies to the other lineages. Basically, D/660 might have been circulated in Italy before its detection, however, those assumptions need to be confirmed by further studies.

This study has some limitations. First of all, the number of tested samples is small, and they belong to swine veterinarians only. In addition, there are no animal samples collected in 2004 in the same geographic area to be tested. One HI‐positive sample was not enough to be tested by the VN assay and consequently, the seroprevalence might be slightly underestimated. The samples were not tested for the presence of antibodies against ICV. So far, serological studies did not detect cross‐reactivity with antibodies directed against human IAV, IBV, and ICV. However, as ICV is a ubiquitous human pathogen, further studies are needed, supported by the development of a virus‐specific assay able to accurately evaluate IDV antibody prevalence in human subjects.^16^, ^23^

Overall, our findings on human serum samples might have two implications. The first one is that undetected IDVs, D/660 and/or D/OK, might have circulated and/or been introduced in Italy as early as 2004 at least in some animal species such as swine. To support these findings and draw definitive conclusions on when IDV has been introduced in Italy and start to circulate, infect, and be transmitted among animals and potentially to humans, it would be important to analyze more archived samples. In particular, samples from animal species susceptible to IDVs infection and from humans, especially people working with animals and those exposed to cattle, covering wide Italian geographic areas. The second one is related to a public health perspective. The data in this study provide further insights on the ability of IDVs to infect and elicit an immune response in humans and should be evaluated considering several aspects. Basically, influenza viruses are characterized by an evolving nature. There is evidence of interspecies transmission and the international appearance of IDV in the animal population worldwide. In addition, IDV can infect ferrets, the gold standard for influenza studies in animals, and guinea pigs, and can replicate efficiently in a human airway epithelium model.^1^, ^15^ Considering all the above and the lesson learned from SARS‐CoV‐2, it would be key not to underestimate the IDV potential as a threat for humans or at least for specific target groups and to continue monitoring IDVs spread in animals and humans.

## FUNDING INFORMATION

Funding information is not available.

## CONFLICT OF INTERESTS

The authors declare that there are no conflict of interests.

## AUTHOR CONTRIBUTION

*Conceptualization*: Claudia Maria Trombetta; *Formal analysis*: Serena Marchi, Fabio Ostanello; *Investigation*: Claudia Maria Trombetta, Serena Marchi; *Resources*: Ilaria Di Bartolo, Fabio Ostanello, Chiara Chiapponi; *Writing—original draft preparation*: Claudia Maria Trombetta; *Writing—review and editing*: Emanuele Montomoli, Ilaria Di Bartolo, Fabio Ostanello, Chiara Chiapponi, Serena Marchi; *Visualization*: Serena Marchi; Project administration: Claudia Maria Trombetta.

## Data Availability

Data available on request from the authors.
